# Comparing Ventilation Parameters for COVID-19 Patients Using Both Long-Term ICU and Anesthetic Ventilators in Times of Shortage

**DOI:** 10.1177/08850666211024911

**Published:** 2021-06-17

**Authors:** Wouter M. Dijkman, Niels M. C. van Acht, Jesse P. van Akkeren, Rhasna C. D. Bhagwanbali, Carola van Pul

**Affiliations:** 1 Department of Intensive Care, 8185 Máxima Medical Center (MMC), Veldhoven, the Netherlands; 2 Department of Applied Physics, Eindhoven University of Technology, Eindhoven, the Netherlands; 3 Department of Clinical Physics, 8185 (MMC), Veldhoven, the Netherlands; 4 Department of Anesthesiology, 8185 (MMC), Veldhoven, the Netherlands

**Keywords:** mechanical ventilation, COVID-19, IC, emergency-ICU, anesthesia-ventilator, closed-loop ventilation

## Abstract

In the first months of the COVID-19 pandemic in Europe, many patients were treated in hospitals using mechanical ventilation. However, due to a shortage of ICU ventilators, hospitals worldwide needed to deploy anesthesia machines for ICU ventilation (which is off-label use). A joint guidance was written to apply anesthesia machines for long-term ventilation. The goal of this research is to retrospectively evaluate the differences in measurable ventilation parameters between the ICU ventilator and the anesthesia machine as used for COVID-19 patients. In this study, we included 32 patients treated in March and April 2020, who had more than 3 days of mechanical ventilation, either in the regular ICU with ICU ventilators (Hamilton S1), or in the temporary emergency ICU with anesthetic ventilators (Aisys, GE). The data acquired during regular clinical treatment was collected from the Patient Data Management Systems. Available ventilation parameters (pressures and volumes: *PEEP*, *P_peak_*, *P_insp_*, *V_tidal_*), monitored parameters *EtCO_2_, SpO_2_*, derived compliance *C*, and resistance *R* were processed and analyzed. A sub-analysis was performed to compare closed-loop ventilation (INTELLiVENT-ASV) to other ventilation modes. The results showed no major differences in the compared parameters, except for *P_insp_*. *PEEP* was reduced over time in the with Hamilton treated patients. This is most likely attributed to changing clinical protocol as more clinical experience and literature became available. A comparison of compliance between the 2 ventilators could not be made due to variances in the measurement of compliance. Closed loop ventilation could be used in 79% of the time, resulting in more stable *EtCO_2_*. From the analysis it can be concluded that the off-label usage of the anesthetic ventilator in our hospital did not result in differences in ventilation parameters compared to the ICU treatment in the first 4 days of ventilation.

## Introduction

During the first months of the COVID-19 pandemic, many patients needed invasive ventilation.^1^ Due to a shortage of ICU mechanical ventilators, many hospitals worldwide needed to deploy anesthesia machines for ICU ventilation (which is off-label use).^2^ In a community effort, guidance was written by scientific societies and experts^2–7^ and manufacturers^8–10^ to ensure safe application of anesthesia machines for long-term ventilation.

Anesthesia machines can apply mechanical ventilation, however the device has an inherently different design. For example in the application of inhaled anesthetic agents a rebreathing system with a scavenging device is used to prevent anesthetic gas entering the environment. Due to variance in intended use, the application of anesthesia devices for long-term ventilation is considered off-label use and is not allowed under normal circumstances. The use of a rebreathing circuit could in ICU-use lead to decreased oxygen levels, increases in inhaled carbon dioxide and more difficult control of the humidity of the applied gas mixture (as the dry fresh gas flow needs to be balanced to the wet circulated exhaled gases).^3^ In addition, the resistance of this circuit is higher and the complete technical setup with safety valves is different, requiring special adaptation for long term ventilation.^2,4–10^ The number of ventilation modes on the anesthesia machines needed to be extended to comply with the requirements for ventilation of COVID-19 patients, targeting a low plateau pressure of not more than 30 cm H_2_O and a higher *PEEP* of at least 10 cmH_2_O.^1^

Not only a shortage of ventilation machines occurred, but alternative locations for mechanical ventilation of ICU patients needed to be found or built. In many cases OR rooms or OR recovery units were selected to be setup as emergency ICU-units. This entailed a fast adjustment of the location to ICU needs, for example technical facilities for gas delivery, room ventilation and availability of all the other medical equipment needed in ICU setting.^5,11–14^

More importantly, extra staff was needed to care for the large influx of patients. The added staff needed to be trained and teams formed for different tasks (ventilation management, fluid management, monitoring, position management). This required a good collaboration and coordination between critical care clinicians, anesthesiologists, critical care nurses, anesthesia workers and newly trained nurses from other units.^1–3,5,14^

Up to now, not many evaluations have been published on the results of application of anesthesia machines in temporary emergency ICUs during COVID-19. Mittel et al^14^ reported the clinical outcome and mortality of patients cared for in their ORICU. And a large multicenter data collection in the Netherlands reported the used ventilation parameters and the clinical short-term outcome with different types of ICUs and OR ventilators.^15^ However, there are limited reports published on the ventilation differences between using an ICU ventilator compared to an anesthesia machine for ICU ventilation in COVID-19 patients. Therefore, the goal of this research is to evaluate the differences in ventilation parameters between the application of ICU ventilation in a traditional ICU setting and the application of ventilation in a temporary emergency ICU using anesthesia machines for COVID-19 patients in the first few days of mechanical ventilation, during the initial phase of the pandemic.

## Methods

### Local Situation During COVID-19 in Phase 1 of the Pandemic

Initially, Máxima Medical Center Veldhoven had an ICU with capacity for 13 patients treated in single patient rooms. All rooms were fully equipped with the necessary medical devices, like ICU ventilator (Hamilton S1), patient monitor (Philips MX800), infusion devices (BBraun). Continuous Veno-Venous Hemofiltration (CVVH), for temporary treatment of patients with acute renal failure was possible on all locations. All the data from these devices was automatically stored in the Patient Data Management System (Philips IntelliSpace Critical Care and Anesthesia ICCA, Philips Medical Systems).

During the first phase of the pandemic, an additional *temporary emergency ICU* was created in the recovery ward of the local OR facilities. Three blocks, each with a capacity of 4 patients were created. These 12 additional beds were equipped with a ventilator (9 with an anesthesia machine (Aisys, GE Healthcare) and 3 with a regular ventilator (Engstrom GE Healthcare)), patient monitor (B650 GE Healthcare), infusion devices (predominantly BBraun, but some other types were also used). All the data from these devices was automatically stored in another Patient Data Management System (HiX, Chipsoft).

### Risk Assessment Before First Use

Before using the anesthesia machines as ICU ventilators, a risk-assessment was performed by a local task-force, consisting of intensive care physicians, an anesthesiologist, technicians, a ventilation practitioner and a clinical physicist. The task-force used the information of the vendor^9^ and the available guidelines at the time (in particular the ASA guideline).^4^ Measures were defined and taken to prevent any significant risk to the patients. Extra attention was given to the equipment configuration. We applied humidification using a Heat Moisture Exchange Filter (HMEF). The exhaled air was filtered to prevent COVID-19. The anesthesia gas scavenging system (AGSS) was opened to atmosphere, as advised by the manufacturer.^9^ The settings of fresh gas flow plays an important role in the ventilation of long-term ICU patients on anesthesia machines. If the flow rate is low, evaporative losses are lower, but moisture may accumulate in the filters and a larger amount of rebreathing may occur. On the other hand, higher flow rate leads to larger evaporative losses but extends the frequency at which the CO2 absorbers need to be changed. We initially used a fresh gas flow of 6-8 l/min according to the guidelines of the manufacturer.^9^ No inhaled anesthetics were used to sedate the patients. The settings of monitoring systems were also adjusted to monitor the gas mixture, in particular the CO_2_.

By implementing these measures and by providing additional training, the benefits of treatment were considered higher than the risks of not treating the patients. This ventilation strategy and work procedure was approved in this emergency situation by both the intensive care physicians, anesthesiologists and clinical physicists as well as the board of directors of the institute, after careful consideration of national^16^ and international guidelines.^1^

### Staffing

During the period that both the ICU and the temporary emergency ICU in the OR were open, the available ICU staff (both nursing and medical staff) was distributed over the 2 locations. In addition critical care caregivers from the anesthesia department, OR and other clinical departments joined the teams. The physicians and nurses all obtained additional training in using the ventilation equipment, by using both video lectures and on-site training facilities. In the period reported in this paper, the ICU nurse to patient ratio was 1:3 and for a short period the ratio was 1:4. Four levels of nursing skills were defined, and the newly added personnel was individually trained on the highest achievable level. As a result, the ICU nursing staff switched from executing to supervising tasks. The physicians underwent a similar process of “dilution” in order to more than double their capacity in a matter of weeks.

Anesthesiologists were trained in the use of ICU devices and respiratory strategies so they could relieve intensivists in daily practice. All anesthesiologists have a base experience on the ICU of minimally 1 year. However, an experienced intensivist remained responsible for the overall treatment. ICU physicians and nurses were trained to use the anesthesia machines. Initiating respiratory support and daily rounds were done by or under supervision of an experienced intensivist. An intensivist was 24/7 available for any respiratory problem.

### Patient Population and Ventilation Strategy

Patients who needed ICU care were distributed over the available patient beds, at first only the ICU unit was used and next, when both units were opened, patients were randomly distributed over the available beds in the 2 units. All the patients that needed ventilation support for COVID-19 treatment gave informed consent to use their data for scientific purposes. Patients with at least 3 days of invasive mechanical ventilation support admitted in either ICU unit in the period 16th March to 30th April 2020, were retrospectively included in the study. The data was de-identified before being processed further. A waiver for this study was provided by the Hospital’s Ethics Committee.

The vast amount of patients presenting in our hospital forced us to transfer some patients to other regions. In the Netherlands the distribution of patients was organized by a regional and national institute so that the patients were evenly spread over the hospitals in the country. This was necessary to evenly share the pressure on the care units. The receiving hospitals were chosen based on capacity and able to provide at least the same level of care. The patients with the lowest transport risk were transferred, this meant that in general the most recently admitted patients were outplaced. As a result, the study population was sicker, and needed more support of vital functions.

The data set consists of 32 patients of which 20 were treated and ventilated in the regular ICU with Hamilton S1 ventilators and 12 in the emergency ICU with Aisys anesthesia machines. Three patients that were treated in the emergency ICU with Engström ventilators were excluded from the analysis as the dataset was too small to be included. The average age, length, body weight and ideal body weight (IBW) of the 32 patients can be seen in Table 1.

**Table 1.** Average Patient Data per Group of Patients Treated With Hamilton Ventilator on ICU or With Aisys Anesthesia Machine on the Emergency ICU.^a^

Average	Hamilton (N = 20)	Aisys (N = 12)	*P*-value	Ham-HPS (N = 8)	Ham-LPS (N = 12)
Age (years)	67	65	NS (*P* = 0.77)	69	64
Length (m)	1.75	1.80	NS (*P* = 0.06)	1.75	1.74
Weight (kg)	90	89	NS (*P* = 0.10)	109	77
Main ventilation modes During first 4 full days	I-ASV (79%), DuoPap (13%) Other (8%)	PCV (63%), PSV (27%)		I-ASV (65%), DuoPap (24%) Other (11%)	I-ASV (88%), DuoPap (6%) Other (6%)

Abbreviations: HPS, High PEEP Strategy; LPS, Low PEEP Strategy; I-ASV, *INTELLiVENT* Adaptive Support Ventilation; DuoPAP, Duo Positive Airway Pressure; PCV, Pressure Controlled Ventilation; PSV, Pressure Support Ventilation.

^a^ For Hamilton subgroups HPS (High PEEP strategy) and LPS (Low PEEP Strategy) were used. I-ASV (*INTELLiVENT* Adaptive Support Ventilation) is a closed loop ventilation mode and DuoPAP = Duo positive airway pressure, both ventilation modes on the Hamilton ventilator. PCV = Pressure Controlled Ventilation. PSV = Pressure Support Ventilation. Both refer to ventilation modes available on the GE Aisys Anesthesia machine.

All patients were treated with a lung protective strategy (tidal volume 6 ml / kg), with a target CO2 resulting in a pH >7.30. PEEP and FiO2 were adjusted to achieve an O2 saturation >92%. If targets weren’t met prone position was applied. All other interventions like the use of NO, epoprostenol and ECMO took place after the 4-day analysis period.

The Hamilton ventilator can ventilate the patients using different modes, of which INTELLiVENT-ASV (I-ASV) closed loop ventilation mode was used as the preferred mode for COVID-19 patients. Intellivent-ASV is an automated ventilation mode which determines the optimal combination of tidal volume and frequency based on calculated values of compliance and resistance and the clinician’s input on target CO2 and SpO2 values. End tidal CO2 and peripheral O2 saturation measurements provide data for a constant feedback loop, used for automated adjustment of Minute Volume, PEEP and FiO2 settings. The algorithms will seamlessly integrate patient triggered breaths with time triggered ones, so the mode can be used for the entire spectrum from paralyzed to completely self-breathing patients. The ventilator will administer breaths in a way that somewhat mimics Pressure Regulated Volume Control and adjusts the applied pressure breath by breath. I-ASV switches back and forth between PCV-like and PSV-like modes.^17^

Measurement faults, triggering difficulties or too large tidal volumes made clinicians in some cases decide to use conventional DuoPap. This is a both time and patient triggered Pressure Controlled ventilation mode. Targeted tidal volume, CO2 and SpO2 values were the same as in the I-ASV group, but now had to be achieved by manual adjustments.

With the Aisys anesthesia machines, the pressure controlled ventilation (PCV) was usually used in the first days of treatment and pressure support ventilation (PSV) was used when spontaneous breathing was possible. A fresh gas flow of 6 to 8 l/min was typically set. Whenever possible a patient triggered ventilation mode was chosen. However sometimes the lung protective strategies required small tidal volumes and paralyzing agents were necessary to facilitate this, resulting in a time triggered mode.

### Data Processing

All the available ventilator parameters were exported from the corresponding Patient Data Management System (PDMS). After de-identification, further processing was performed using Mathematica version 12.1. The Aisys data was stored at high temporal resolution, without being validated by nurses. Whereas the Hamilton data was only stored after validation by nurses in the 2-hourly nursing round. Therefore, time resampling (not time averaging) to 2-hourly data is performed for each patient using a “TimeSeriesResample” method in Mathematica. If there was no data available close to a 2-hour point, the value was reported as “missing” and excluded in the calculation of averages for that timepoint. This can occur as not all parameters are measured in all possible ventilation modes used. In addition, all time information was stored as time from first moment in the unit. For each patient, both a daily average of the parameter and an average for the first 4 full days of stay were calculated. Longer time periods could not be evaluated as around 30% of all included patients were moved to a different hospital after 5 days due to the high influx of new patients. The compliance and resistance were measured continuously on both ventilators. However on the Hamilton ventilator, the so-called static compliance Cstat is calculated using a least squares fitting method^18,19^ and on the Aisys system a dynamic compliance (Cdyn) is determined. Cstat can only be reliably used if there is no spontaneous breathing, therefore for further processing only Cstat is used if the spontaneous breathing frequency was 0. For the Aisys machine, the spontaneous breathing frequency was not stored and therefore the continuously measured Cdyn could not be used for further processing.

Thereafter, for a sub-analysis, the data was split into 2 different groups based on the *PEEP*-value at start. Initially, a high-PEEP was used based on ARDS protocols. However, clinical observations and later also literature^16,20^ suggested the need to lower the PEEP values. In the first week all our patients could still be treated in the regular ICU, therefore this high-*PEEP* strategy group (HPS, average value >14.5 cm H_2_O for the first 4 days) only involved 8 Hamilton treated patients. For all other patients a lower-*PEEP* strategy is used, called the LPS group).

For comparison of the tidal volume *V_tidal_* we use the correction for ideal body weight. To convert the tidal volume of Aisys to the tidal volume per ideal body weight, the tidal volume has to be divided by the ideal body weight of the patient using the following equations as used in Hamilton ventilators, for adult male: IBW (kg) = 0.9079 × Patient height (cm) − 88.022 cm × kg/cm and adult female: IBW (kg) = 0.9049 × Patient height (cm) − 92.006 cm × kg/cm.^18^

### Statistics

Statistical analysis was performed using SPSS Statistics version 25 (IBM corporation, 2017). To investigate the ventilation parameters between the Hamilton treated patients and the Aisys treated patients, we focused on the parameters that were available for most ventilation modes (*PEEP*, *P_insp_*, *P_peak_*, *V_tidal_*, *EtCO*_2_, *SpO_2_*). First, we compared all patients in the Hamilton group to the patients in the Aisys group. Then, we performed a sub-analysis on the patients treated with LPS strategy and compared again the ventilation parameters between Hamilton and Aisys. In addition, a second sub-analysis was performed for patients who were treated for more than 90% of the time in the first 4 days with the closed-loop ventilation I-ASV, compared to patients (n = 5) who were less than 60% of the time treated with I-ASV. To compare these parameters between the 2 groups, for all mentioned analyses, we used an independent samples Mann-Whitney U-test. A *P-value <*0.05 was considered significant.

## Results

### Comparing Ventilators

**Figure 1.**
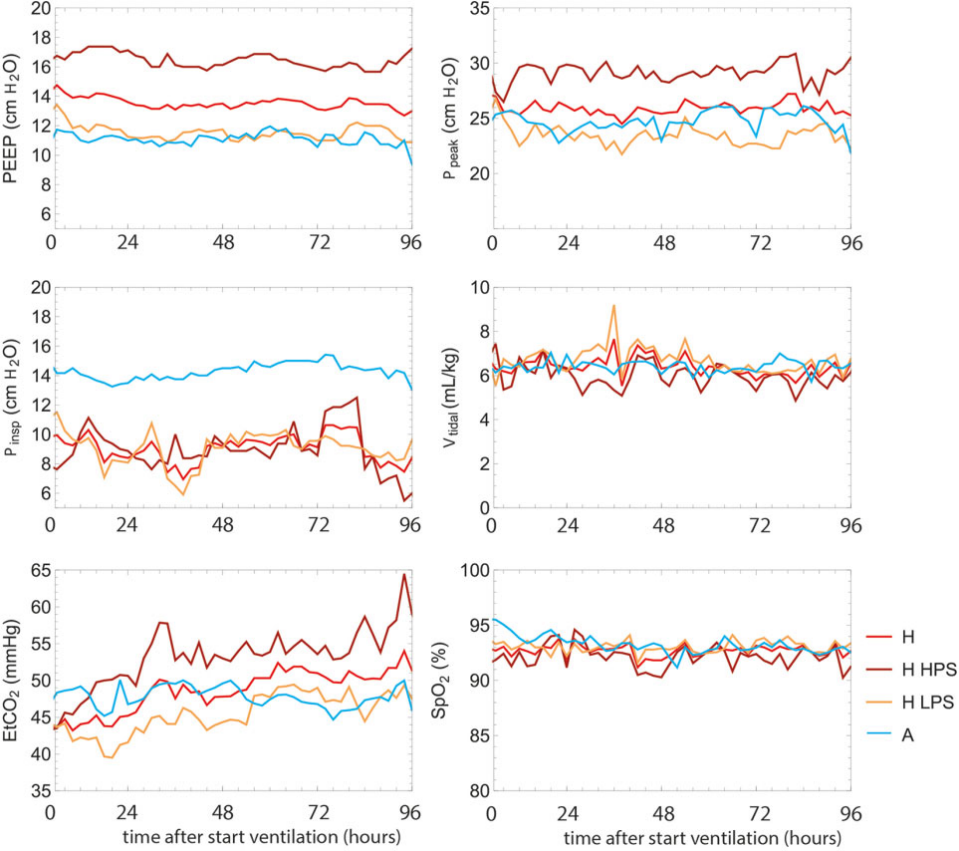
Average of all patients per group for PEEP, Ppeak, Pinsp, Vtidal, EtCO2 and SpO2. The colors indicate the different groups evaluated: patients treated with Hamilton S1 (total n = 20) for both the High (HPS, n = 8) and Low PEEP Strategy (LPS, n = 12) and the Aisys Anesthesia machine (n = 12).

For the first 4 full days of ventilation, on Hamilton devices the mode I-ASV was used in 79% of the time, DuoPap 13% of the time and other ventilation modes, like ASV without INTELLiVENT, the rest of the time. For the Anesthesia machines, PCV mode was used 63% of the time and PSV mode the remaining 27% of the time. The average value for the 2-hour resampled timepoints for the parameters *PEEP*, *P_peak_*, *P_insp_*, *V_tidal_*, *EtCO*_2_ and *SpO_2_* of all patients are displayed in Figure 1, for both the full Hamilton (H) and Aisys (A) groups and the subgroups HPS and LPS. The average values for 4 days are shown in Table 2.

**Table 2.** For Each Ventilation Related Parameter, the 4-Day Average per Group of Patients Are Shown, for Patients in the Hamilton (H) and Aisys (A) Groups and the Subgroups HPS and LPS.^a^

Average of 4 days	Unit	H (n = 20)	HPS (n = 8)	LPS (n = 12)	A (n = 12)	*P*-value (H vs A)	*P*-value (H*P*S vs LPS)	*P*-value (LPS vs A)
PEEP	cmH_2_O	13.5	16.4	11.5	11.2	*P* = 0.024	*P* = 0.001	NS
P_peak_	cmH_2_O	25.7	29.2	23.3	24.9	NS	*P* = 0.004	NS
P_insp_	cmH_2_O	9.0	9.1	8.9	14.0	*P* = 0.000	NS	*P* = 0.002
V_tidal_	mL/kg	6.5	6.0	6.8	6.5	NS	*P* = 0.025	NS
EtCO_2_	mmHg	47.6	52.5	44.4	47.7	NS	NS (*P* = 0.06)	NS
SpO_2_	%	92.8	92.2	93.2	93.3	NS	*P* = 0.004	NS
C	ml/cmH_2_O	39.6	41.8	37.2	—	—	NS	
R	cmH_2_O/L/s	11,8	11.2	12.3	—	—	NS	
F_i_O_2_	%	0.51	0.56	0.48	0.47	NS	*P* = 0.010	NS

^a^ The *P*-value for statistical testing between the groups is shown in the last 3 columns.

For the *PEEP*, the high value for the HPS group is characteristic. In general, for the LPS group (n = 12), the data shows a striking resemblance with the Aisys (n = 12) for most parameters, except for the lower *P_insp_* and the seemingly lower *EtCO*_2._ As can be expected, pressures in the HPS group are higher than in the LPS and Aisys groups and therefore the average for the Hamilton group (H), containing the information of all 20 patients, is on average influenced by the HPS group.

**Figure 2.**
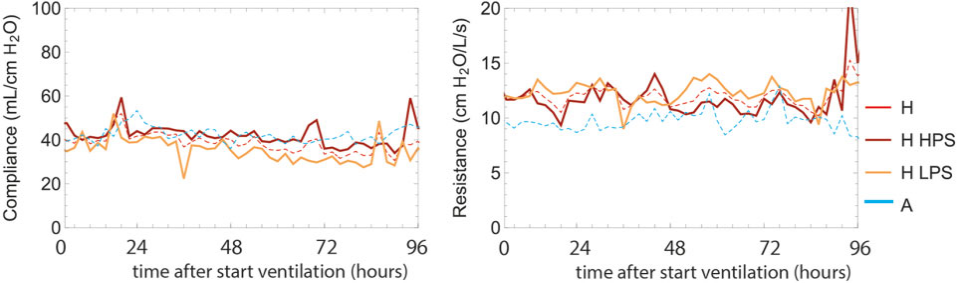
Compliance and resistance (Rinps) compared for Hamilton S1 (All H, LPS and HPS groups) and Aisys (A) ventilated patients. The compliance calculated on the Aisys is a dynamic compliance. The compliance for the Hamilton is calculated using the LSF method for continuous determination based on measured pressures and volumes, and is only used in the analysis on those moments that there is no spontaneous ventilation.

Though large differences are observed, in particular between LPS and HPS in pressures and *EtCO_2_*, there are no striking differences in Compliance and Resistance (Figure 2). Although in the tidal volume a small, but significant lower value for HPS is observed compared to LPS.

Statistical testing (Table 2) showed significant differences for *P_insp_* and *PEEP* between the 2 ventilators. The difference in *PEEP* only exists if the HPS group is included, as no significant difference can be observed if only the LPS group is compared to the Aisys group. Major differences are observed between the LPS and HPS ventilation strategies, in particular in pressures but also in *SpO_2_* and *V_tidal_*.

### Closed Loop Versus Non Closed-Loop

**Figure 3.**
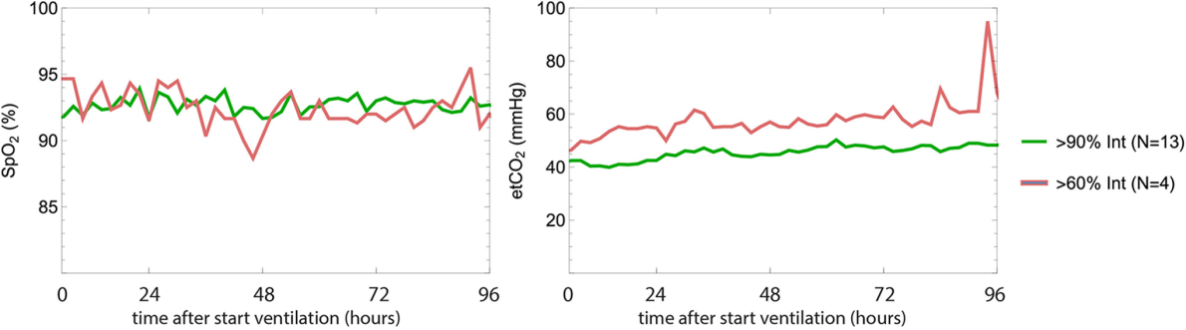
EtCO2 and SpO2 as a function of time from start of ventilation for the high INTELLiVENT-ASV and low INTELLiVENT-ASV groups.

The average *EtCO*_2_ and *SpO_2_* values for the Hamilton patients were separated into 2 groups: patients treated mainly with I-ASV (named the HI-group, more than 90% of the time; n = 13) and a group of patients for whom I-ASV was used in less than 60% of the time (n = 4, named the LI-group). The results in Figure 3 show that in the first days of treatment, *EtCO*_2_ values were lower (*P* = 0.23) in the HI-group than in LI-group, whereas the measured peripherally measured saturation *SpO_2_* did not differ. We also found a difference in airway resistance between the 2 groups (R is lower in the >90% group, *P* = 0.006).

**Figure 4.**
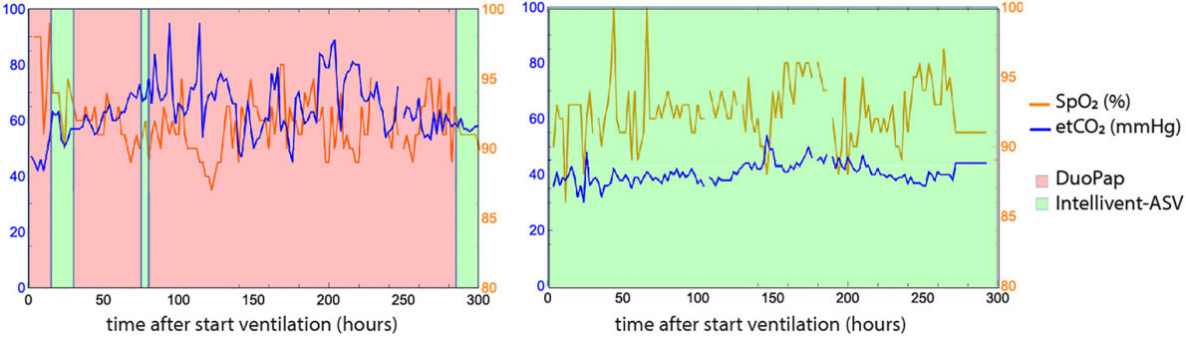
EtCO2 and SpO2 over time of a patient with 23%, 53% and 98% INTELLiVENT-ASV usage. The background color shows whether or not INTELLiVENT-ASV is used at that time. In this graph a longer timescale is used (300 hours) in order to fully show the differences.

In Figure 4, 2 patients are shown to display the fluctuations in *EtCO*_2_ and *SpO_2_* at patient level, comparing I-ASV to another ventilation mode. On the left-hand side, the patient was treated with I-ASV in 23% of the time. On the right-hand side, another patient with 98% I-ASV, showing the *EtCO*_2_ to be more constant during I-ASV treatment.

## Discussion

Our results show that ventilation parameters are similar in the first 4 days for patients treated with an ICU ventilator and with an anesthesia machine, except for P_insp_. This is important because this suggests that with the adjustments as advised by the community,^4^ similar ventilation treatment was obtained in this emergency situation using anesthesia machines for ICU ventilation. Analysis of ventilation parameters for more than 4 days was not possible as in both H and A groups, around 30% of the patients were transferred to hospitals in other regions. The choice which patients would be outplaced was based on the transport risk. The cohort of patients not eligible for outplacement was thought to have a higher transport risk, for instance because of prone position or the need of higher FiO2 or PEEP. Because of this outflux of patients we could only analyze the first 4 days of admission. Analysis of a longer period would have meant limiting the study group to the non-transferred patients, making it too small. In this cohort, application of an anesthesia machine for a prolonged period of time on patients with severe respiratory insufficiency (classified as off-label use) did not introduce any extra risks. It proved to be beneficial in expanding the ventilation support capacity in a time of absolute shortage. Only one incident occurred when an anesthesia machine failed due to a technical issue unrelated to prolonged application. A backup ventilator was used to maintain ventilation support. In line with other reports, some problems with filters and humidity in the ventilation tubes of the anesthesia machines have been reported, as was anticipated beforehand.^4^ The average values measured for the ventilator parameters are similar for most measured parameters and are mostly in line with the observation in the Dutch multicenter study, in which MMC participated as one of the 18 ICUs.^15^

The difference in *P_insp_* can be explained by the difference in definition of this parameter between the 2 machines. For most patients on the Hamilton ventilator the I-ASV is used, in which mode *P_insp_*, is the automatically calculated target pressure, according to the operator manual of the Hamilton S1.^18^ With the Aisys anesthesia machine, the parameter *P_insp_* is the set inspired pressure that is logged.^21^ Another possible explanation is that spontaneous breaths in I-ASV mode occur more often and they lead to lower *P_insp_*. However, the information on spontaneous breaths in the Aisys is not logged, refraining us to further investigate this hypothesis.

The effects of using a different *PEEP* strategy are more pronounced than the effects of using an anesthesia machine. Our dataset contains some patients that were treated with high *PEEP*, as that was the advised PEEP setting in the first phase of the crisis. As clinical experience with COVID-19 increased, this strategy was changed to lower *PEEP,* in line with research showing that this had better results.^20^ We did clinically observe differences in compliance between the LPS and HPS in individual cases, however we cannot confirm this in statistical testing between these 2 groups. Research by Roesthuis et al suggests that the decrease of compliance in response to high *PEEP* seems to indicate that COVID-19 lesions are not recruited and the decreased compliance is a result of hyperinflation.^20^ The compliance we observed in our population is somewhat higher than the compliance in the multi-center study in the Netherlands,^15^ perhaps due to the fact that most patients in our center were treated with the low-*PEEP* strategy. In addition, if values for dynamic compliance are also included in the national analysis, the different method of determining continuously static compliance based on LSF method^18,19^ may also explain our higher value as Cstat is usually higher than Cdyn. The high PEEP group had a lower tidal volume despite a similar *P_insp_*, and according to Table 2 no significant difference in compliance. However, tidal volumes are measured continuously, during periods with spontaneous breathing and periods without, whereas the compliance is only evaluated if the spontaneous breathing frequency is zero. The periods with spontaneous breathing can affect tidal volumes as measured. In addition, patients in the LPS group had more periods of spontanenous breathing than patients in the HPS group.

In our intensive care unit using Hamilton ventilators, closed loop ventilation is used most of the time, indicating that also in COVID-19 patients this ventilation mode is very useful, although not suitable for all patients. I-ASV mode relies heavily on peripheral measurements of *S_p_O_2_* and *EtCO_2_*. In COVID-19 patients the gap between the end tidal and actual PCO_2_ often proves to be too large for safe application of I-ASV. Measurement failure with low *SpO_2_* values will cause a rapid increase in FiO_2_ to prevent hypoxia. This will make *SpO_2_* levels rise, in spite of the measurement fault after which the FiO_2_ is slowly decreased again. The result is a repetitive pattern of increasing and decreasing FiO_2_. In addition, instability in the *EtCO_2_* was observed. Both could lead to an oscillating pattern, that necessitates abortion of the I-ASV closed loop application to be replaced with a less automated mode. Another observation leading to aborting the I-ASV mode was the slower responses in the closed loop system needed for COVID-19 patients. Nevertheless, in most patients the I-ASV mode functioned well because of the ability of I-ASV to constantly monitor the patient and adapt its settings to the patient’s condition resulting in a more constant EtCO_2_.

There are several limitations to our study. First of all, the number of patients included in our study is quite low, as fortunately the use of additional anesthesia machines was only required for a short period during the pandemic. Also in later phases, like last winter, there was no more shortage as more Hamilton ventilators were available that had been commissioned in Spring 2020. In addition, only ventilation parameters in the first 4 full days of treatment could be used, as many patients were transferred to other hospitals because of the lack of capacity at the high rate of influx. Therefore, differences occurring in later stages could not be evaluated.

Second, there may have been a selection bias between patients treated in the ICU compared to patients treated in the temporary ICU, as in the ICU itself full dialysis is possible. It may be that more ill patients are included in our ICU patient group, although on both ICU units CVVH was available and often used in both units. However, in the observed period due to shortage there was no time for further selection and therefore we consider the contribution of this bias to be small.

Third, we would like to stress that comparing 2 different ventilators, using different ventilations modes, different pressure sensors in different locations of the ventilation circuit, different S_p_O_2_ sensors, side stream versus mainstream *EtCO_2_* measurements and also different methods to continuously estimate the compliance and airway resistance, should be done with caution. However, in these difficult times this was the available information used to guide clinical decisions continuously.

Fourth, the assumption was made that all COVID-19 patients have the same disease pattern, though in literature it is suggested that different types of COVID-19 may occur^22^ and different time paths may occur.

Fifth, the Hamilton dataset contained low temporal data only, as the data is validated by the nurses before it is added to the dataset. This makes the data more reliable, but the low temporal resolution prevents a comparing analysis with more condensed datasets.

As little was known about the treatment of COVID-19 patients at the time of this study constant adjustments were made in treatment strategies. For example, this data set contains some patients that were treated with the high *PEEP* strategy. Later, after more clinical experience with COVID-19 patients, this strategy was changed to low *PEEP,* in line with research from the first phase of the pandemic, showing that this had better results.^20^ Changes in medical treatment during this period were possibly larger than the effects by using a sub-optimal ventilator.

## Conclusion

We observed little difference between the ventilation parameters in patients in the first 4 days of mechanical ventilation using an ICU ventilator (Hamilton) and using an anesthesia machine (Aisys). In future emergency situations where there is a shortage of ventilators, we may again use anesthesia machines for long-term mechanical ventilation.
